# Effectiveness of spaced repetition for clinical problem solving amongst undergraduate medical students studying paediatrics in Pakistan

**DOI:** 10.1186/s12909-024-05479-y

**Published:** 2024-06-18

**Authors:** Shazia F. Durrani, Naveed Yousuf, Rahila Ali, Fatima Fakhir Musharraf, Ammara Hameed, Hussain Ahmed Raza

**Affiliations:** 1 Department for Pediatrics, Bahria University Medical & Dental College, Karachi, Pakistan; 2 Department for Educational Development, The Aga Khan University, Karachi, Pakistan; 3 Jinnah Sindh Medical University, Karachi, Pakistan

**Keywords:** Spaced repetition, Medical education, Learning

## Abstract

**Background:**

Studies using spaced repetition for teaching and learning in undergraduate clinical rotations such as paediatrics are limited, even more so in the South Asian region. Therefore, this study aimed to identify the effectiveness of utilizing spaced repetition compared to traditional learning methods among undergraduate medical students during their paediatric rotation at a medical university in Pakistan.

**Methods:**

Bahria University Medical and Dental College (BUMDC) conducted quasii-experimental research in Karachi. Four topics were identified from the Year 5 Pediatrics curriculum to be used in the study, using which the study content was developed along with 50 multiple choice questions (MCQs) for assessment. All BUMDC Year 5 medical students rotating in Pediatrics were included and randomly allocated to the control or intervention group. In the control group, they provided the students with traditional study methods consisting of books and lectures to learn topics. In the intervention group, we created an Anki flashcard deck of the same topics to enable learning via spaced repetition. The researchers conducted a pretest and post test assessment of the 50 MCQs in both groups at the beginning and after the four-week study interval. The data were analysed using SPSS 19.

**Results:**

A total of 115 BUMDC medical students agreed to participate in the study; 70 (59.1%) were in the intervention group, and 45 (41.7%) were in the control group. The pretest mean score of the control group was 27.96 ± 3.70, and the posttest mean score was 27.22 ± 5.02, with no statistically significant difference at the 95% confidence level. The mean score of the pretest for the intervention group was 27.93 ± 4.53, and that of the posttest was 30.8 ± 4.56, with a statistically significant difference at the 95% confidence level. The intervention showed a significant effect size of 0.8.

**Conclusion:**

The use of spaced repetitions resulted in significantly greater scores for medical students studying paediatrics than for those using more traditional methods of learning, compromising medical books and lectures. Considering that medical students need to retain a vast amount of information, using spaced repetition through flashcards can be a more effective learning tool that is more cost-efficient and time-efficient than traditional learning methods.

**Supplementary Information:**

The online version contains supplementary material available at 10.1186/s12909-024-05479-y.

## Background

Learning in medical school comprises building factual and theoretical knowledge, followed by practical application of that knowledge in a clinical setting. Due to the extensive medical curriculum, undergraduate medical students need help in the long-term retention of knowledge taught in clinical and nonclinical disciplines. Furthermore, medical students are in critical need of efficient self-study teaching and learning methods to help them retain the vast amounts of knowledge and clinical skills required to fulfil medical school competencies [1]. However, students must be aware of efficient learning strategies that can improve their long-term memory retention [2].

A study by Gilbert, M. M. (2023), conducted in the Boonshoft School of Medicine, USA, explored techniques that help students improve learning and found that spaced repetition was the best technique for enhancing student learning [3]. It has already been proven that the retention of factual knowledge is improved by using practice tests and spaced repetition [4]. Undergraduate and postgraduate medical students globally use spaced repetition despite being a comparatively new concept in medical education. It not only helps individuals attain proficiency but also aids in active recall and the application of clinical knowledge [5]. Numerous studies have shown evidence of the effectiveness of spaced repetition in long-term memory retention. A study of undergraduate medical students showed better short-term and long-term memory retention using spaced repetition learning [6].

Flashcards are the most popular method for utilizing spaced repetition learning. Anki. Web consists of flashcard software built on a spaced repetition algorithm. The literature has shown that Anki flashcards are beneficial for learning basic sciences such as anatomy [7]. Anki has become a popular learning tool in medical schools across the globe since it not only boosts performance on medical licensing exams but also allows most American first-year medical university students to use Anki flashcards to supplement their studies [8, 9].

In particular, medical students struggle to learn about numerous volatile topics that require quick recall, such as developmental milestones, immunizations, and integrated management of neonatal and childhood illnesses. Due to difficulty in recalling such minute facts, students will undoubtedly need help to perform well in their paediatric rotations. Furthermore, medical educators have conducted global studies to identify the most effective teaching and learning strategies in medical education. However, data about South Asia, such as Pakistan, still need to be available. Additionally, researchers have yet to perform many studies to determine effective teaching and learning strategies in paediatric rotations for undergraduate medical students. Therefore, this study aimed to identify the effectiveness of the spaced repetition learning strategy compared to traditional learning methods for undergraduate medical students during their paediatrics rotation at a private medical university in Pakistan.

## Methodology

### Setting and participants

This quasi experimental study was conducted at Bahria University Medical and Dental College (BUMDC) in Karachi, Pakistan. The total duration of the study was six months, and participant assessments were performed at four-week intervals.

The sample size was the whole population, which included the whole class of BUMDC year 5 medical students rotating in paediatrics. Purposive sampling, where total population sampling was performed.

**Table:** 

Sample Size for Frequency in a Population						
Population size(for finite population correction factor or fpc)(*N*):					150	
Hypothesized % frequency of outcome factor in the population (*p*):					80%+/-5	
Confidence limits as % of 100(absolute +/- %)(*d*):					5%	
Design effect (for cluster surveys-*DEFF*):					1	
**Sample Size(** ***n*** **) for Various Confidence Levels**						
	**Confidence**	**Level(%)**	**Sample Size**			
	95%		94			
	80%		63			
	90%		81			
	97%		101			
	**99%**		**112**			
	99.9%		124			
	99.99%		131			
Equation						
Sample size ***n*** = [DEFF*Np(1-p)]/[(d^**2**^**/Z**^**2**^_1−α/2_*(N-1) + p*(1-p)]						

The inclusion criteria included all BUMDC Year 5 medical students rotating in Pediatrics. The exclusion criteria consisted of five medical students who did not have internet access or who did not consent to participate in the study. The students were randomly divided into two groups: the intervention group and the control group. Each group had an equal number of students who were selected randomly.

### Ethical approval

Data collection started after receiving approval from the Ethical Review Committee (ERC) of AKU (2021-6175-19040) and BUHSC (69/2020).

### Consent from participants

The AKU-approved consent form was printed, and informed consent was obtained from five medical students of the BUMDC who agreed to participate in the study. Students were informed that their names and results would not be disclosed to anyone (except the students themselves) and that the results would not affect their rotation performance scores. Upon agreement, the students were asked to sign the form that the researcher and principal investigator had already signed. Copies of the form were given to the students after the signatures were completed. The forms are attached as additional file 5.

### Materials

#### Content selection and validation

Four topics were identified from the year 5 pediatrics curriculum to be used in the study for assessing learning in both the intervention and control groups. The topics included developmental milestones, Integrated Management of Neonatal and Childhood Illnesses (IMNCIs), immunizations, and malnutrition. These topics were selected since knowledge of these topics is commonly applied in general paediatric clinics. Furthermore, they encompass a broad range of crucial factual knowledge that is difficult to recall during clinical practice. The learning objectives of these four topics, along with a table of specifications (ToS) with relative weights assigned to each topic for pretest and post test assessments, are provided in additional annexure file 1.

We identified eight experts who were FCPSs trained or equivalent in pediatrics, had at least five years of teaching experience, and had advanced qualifications in medical education (master’s or advanced diploma or equivalent). All eight experts were sent a request through email to be a member of the expert validation panel for our study to review our learning objectives and weight given to each topic and to select the pretest and post test multiple choice questions (MCQs) for assessment. Five experts responded positively and were included after their approval to participate. The five experts were requested via email to review and rate their agreement with the learning objectives and the weights assigned to each of the four topics we selected from the year 5 Pediatrics curriculum using a structured form with a four-point scale that varied from ‘strongly disagree’ to ‘strongly agree’ (Annexure III). All five experts agreed on the topics’ learning objectives and relative weights (file I). Any minor suggestions provided by the experts were reviewed and incorporated as necessary (Annexure IV).

#### Spaced repetition and mass learning materials

Anki is a flashcard software built upon a spaced repetition algorithm that allows users to review studied cards after one day, three days, seven days, 14 days, and 28 days. Flashcards are digitally made and appear at intervals depending on user recall. Flashcards are organized into decks, and users review these decks regularly. Based on the algorithm, the card being studied can be a new card not previously created by the user or an older card that reappears after an appropriate interval. Reviewed Anki cards that are difficult to remember reappear after a short interval, whereas quickly recalled cards reappear after a longer interval. This algorithm effectively uses spaced repetition technology and allows users to customize their learning based on their recall power. This spaced repetition learning method ensures revision of all topics provided on the flash cards.

A deck of Pediatrics flashcards was created using the learning objectives of the four selected topics. The flashcards were sent to the expert panel for review and approval. The content of the flashcards was developed using year five medical course books, lectures, and guidelines, with references to their respective sources included. The flashcards were made on the Anki website and were sent to the students in the intervention group. Instructions were given to the students in the intervention group on how to use the Anki website, along with step-by-step instructions on how to use the Pediatrics flashcard deck. If they had any additional queries, the students were also provided with support on WhatsApp regarding how to use the Anki flashcards. Upon request, a few students were guided face-to-face using the Anki website and flash cards.

The control group was requested to follow traditional learning methods used at the university, which consisted of using lecture notes and books that covered the same learning content provided in the flashcards given to the other group.

To eliminate potential confounders and correct the selection of students, intervention group students had to enrol on the Anki website using their emails. The flashcards and download instructions, including the paediatric flashcards deck, were given only to the students in the intervention group, who were given instructions to keep them private. Both groups were added to the respective WhatsApp group chats for daily feedback. This feedback ensured that the intervention group had no trouble using the new Anki flashcards. The total study time given to both groups was four weeks to ensure that all topics were studied thoroughly by both groups, and enough time was given for learning and retaining the provided information.

### Development and validation of assessment questions

Seventy-five one-best multiple-choice questions (MCQs) were developed based on the learning objectives of the four topics selected. Particular attention was given to ensuring that all questions not only required factual recall but also involved critical thinking for problem-solving and applying objective knowledge.

A tool for MCQ validation was created for the expert panel to use, consisting of a four-point rating scale for each item assessing the level of agreement (strongly disagree, disagree, agree, strongly agree) based on relevance, content, and clarity. This tool was sent to the expert panel through email for review and validation. After incorporating expert feedback, 50 MCQs were approved and finalized for the pretest and post test assessments. These 50 questions were also validated according to exam weight and learning objectives.

### Data collection

The Quizizz online quiz website (quizizz.com) was used for the pretest and post test assessments in both intervention and control groups, which consisted of the 50 validated MCQs.

Participants and faculty members were informed of the date, location, and timing of the pretest assessment along with test instructions via email. On the day of the pretest assessment, the students were asked to sign an attendance sheet and sign up for the test using their names and roll numbers. The pretest was administered through the Quizizz website. Each MCQ was given 90 s to attempt, leading to 1 h and 15 min for the pretest assessment of 50 MCQs. Four faculty members were involved in the invigilation to ensure fair conduct of the examination.

After four weeks of study, a posttest assessment was performed. The participants and faculty members were again informed of the date, location, and timing of the post test assessment along with the test instructions via email. The post test assessment was the same as the pretest assessment. Similarly, each item was given 90 s, and 1 h and 15 min were given for the entire post test. On the day of the post test assessment, the students were told to sign an attendance sheet and sign up for the post test assessment by name and roll number. Four faculty members were involved in the invigilation to ensure fair conduct of the examination.

### Ethical considerations

Student names were kept anonymous since coding was used to mask their names. No one had access to the data except the data reviewers. All hard copies of the consent forms were kept in a locked file, and the assessment results were password-protected on a computer. Both assessments were formative and did not affect their rotation performance assessment. After the post test assessment was completed, the Anki flashcard deck and instructions were provided to all the students, including those in the control group, in case they wished to use them later to prepare for their summative assessment.

## Data analysis

The Statistical Package for Social Sciences (SPSS) version 19 was used to analyse the data. All inferential analyses were two-sided, and *p* values < 0.05 (95% confidence level) were considered to indicate statistical significance. For pretest and post test assessment results, quantitative data are reported as the mean ± SD, whereas for qualitative variables, frequencies are reported as percentages. Content validity evidence (ToS) was obtained from content experts (Appendix V). Reliability was determined using Cronbach’s alpha for both pretest and posttest assessments.

**Fig. 1:**
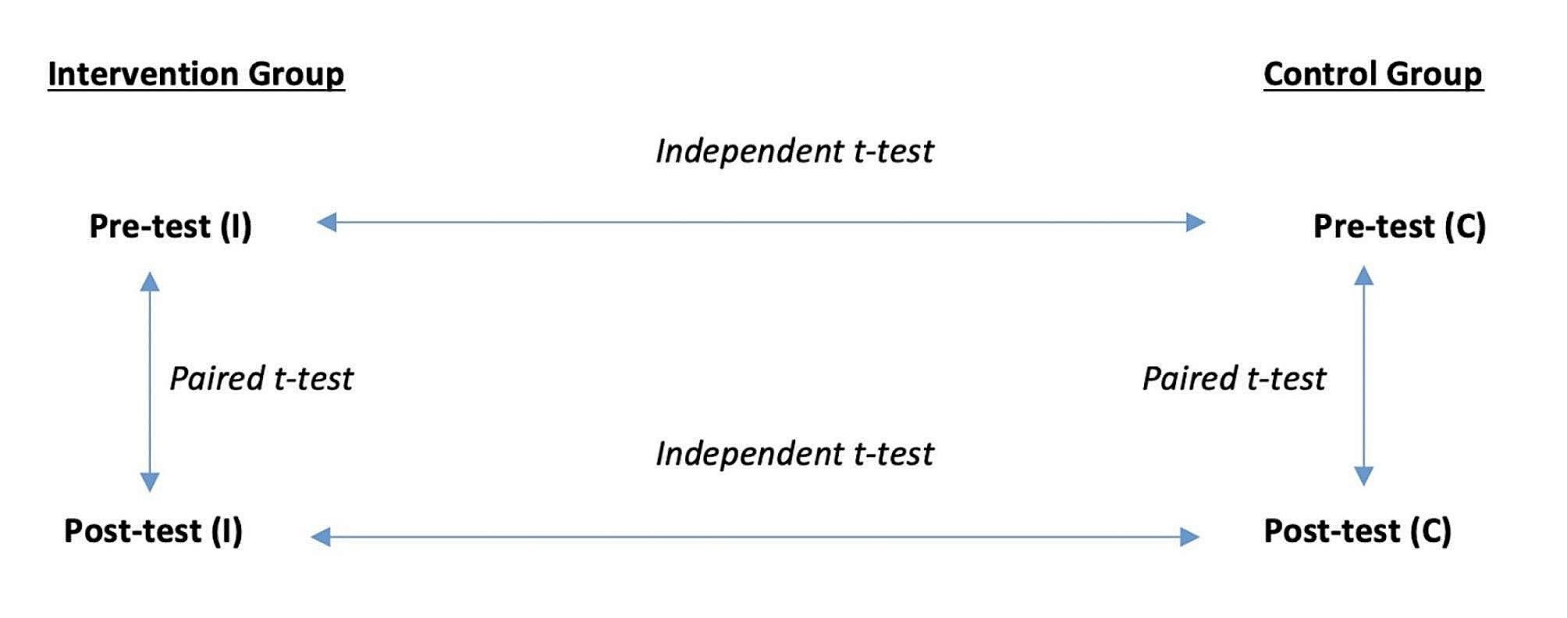
Plan for inferential analysis

An independent sample t test was used to determine the difference between the two groups (intervention versus control) between the pretest and posttest assessments. A paired t test was used to independently measure the differences between the pretest assessment results and posttest assessment results of the two groups (intervention and control). Figure 1 depicts the inferential strategy utilized.

## Results

Of 145 year 5 BUDMC medical students, 115 agreed to participate in the study for pretest and posttest assessments. Participant characteristics and test results are shown in Table 1. Among these, 45 (41.7%) were from the control group, and 70 (59.1%) were from the intervention group. Overall, among all participants, 77 (65.8%) were female, and 40 (34.2%) were male.

**Table 1:** Comparison of participant characteristics and test results between the control and intervention groups

Test Results	All Participants (*n* = 115)	Control Group (*n* = 45)	Intervention Group (*n* = 70)	*p* value
Pretest (Mean *±* SD)	27.94 *±* 4.18	27.96 *±* 3.69	27.93 *±* 4.53	*p* = 0.973
Posttest (Mean *±* SD)	29.41 *±* 5.04	27.22 *±* 5.02	30.86 *±* 4.56	*p* < 0.001

*Note: p value significant < 0.05*

Overall, for all participants, the mean pretest score was 27.94 ± 4.185, while the posttest score was 29.41 ± 5.04. There was no significant difference in the pretest scores between the intervention and control groups (*p* = 0.973). However, a significant difference was found between the posttest scores between both groups, with the intervention group scoring higher, with a mean score of 30.86 ± 4.56, while the control group scored lower, with a mean score of 27.93 ± 4.53 (*p* < 0.001).

A comparison of the pretest and posttest results using a paired t test is shown in Table 2. Overall (need info for overall if there was a significant difference). For the control group, there was no significant difference between the pretest and posttest scores (*p* = 0.275). However, for the intervention group, the posttest score was significantly greater, with a mean of 30.86 ± 4.56, than the mean pretest score of 27.93 ± 4.53 (*p* < 0.01). The reliability analysis using Cronbach’s alpha for the pretest was low (0.466), while for the posttest, it was high (0.717).

**Table 2:** Comparison of pretest and posttest results within participant groups

Participants	Pretest Result (Mean +/- SD)	Posttest Result (Mean +/- SD)	*p* value
All Participants (*n* = 155)	27.94 *±* 4.18	29.41 *±* 5.04	-
Control group (*n* = 45)	27.96 *±* 3.69	27.22 *±* 5.02	0.275
Intervention group (*n* = 70)	27.93 *±* 4.53	30.86 *±* 4.56	**< 0.01**

Note: *Note: p value significant < 0.01*

## Discussion

Effective learning and recall of essential pediatric medicine concepts to perform well in a clinical setting is vital for medical students. Educators and students employ various teaching and learning methods to enable efficient learning and recall of complex concepts. Our study showed that compared to students in the traditional learning methods group, students in the intervention group using spaced repetition performed significantly better on their posttest assessments than on their pretest assessments. Although statistically, posttest scores showed high reliability, pretest scores demonstrated low reliability, which may be due to no or minimal preparation of the students for the pretest assessment.

Our study demonstrated the efficiency of using Anki flashcards for spaced repetition as an effective tool for learning pediatric medicine topics compared to traditional learning methods. Our study revealed that not only does space repetition lead to more effective learning, but students also tend to score higher due to this learning method. A recent blog by Lecturio, a commonly used study platform by medical students, agrees with our findings and states that using flashcards leads to active recall of knowledge, which has been shown to be more advantageous in retaining information than other passive learning methods [10]. Compared to basic sciences subjects of medicine, which require extensive studies of minute factual details, several studies have also shown flashcards to be practical learning tools for retaining vast amounts of factual knowledge [7, 11, 12]. Interestingly, Lu et al. also found that using Anki flashcards for spaced repetition resulted in higher scores on the United States Medical Licensing Exam (USMLE) Step 1 exam, which is heavily based on fundamental scientific factual knowledge [13].

Similarly, in clinical fields requiring more application of knowledge than just factual recall, the use of flashcards has also been shown to improve performance scores and retention, such as in the fields of obstetrics and gynaecology, radiology, psychiatry, urology and otolaryngology and internal medicine [14–19]. With respect to the field of pediatrics in particular, most studies performed to show the effectiveness of spaced repetition have been conducted mainly at the postgraduate and professional levels. For instance, in contrast to our study findings, a study performed on pediatric residency trainees by McConnery et al. using spaced repetition learning did not show improved performance on assessment [20]. However, they noted that barriers such as limited time and limited participation may have hampered learning. Similarly, in another study related to pediatric acute illness management, after observing 12 critical illness scenario demonstrations, which were spaced fortnightly, learners demonstrated improved pediatric resuscitation skills [21].

Most studies on spaced repetition tend only to assess direct recall, especially those performed with students from undergraduate medical schools. In comparison, our study made a conscious effort to evaluate students not only for factual recall but also for problem-solving and clinical application of knowledge. Only a few studies in the literature have also assessed the problem-solving skills of students at the undergraduate level. One such study by Tshibwabwa et al. was conducted in second-year preclinical students at the American University of Medicine, in which they used spaced learning in radiology as part of an integrated curriculum. As a result, spatial learning enhances the retention of radiological concepts and improves problem-solving and radiological assessment skills [22].

Furthermore, with the South Asian region under the spotlight, we found very little research on spaced learning methods in medical education. Of the few studies we observed from the region, two were performed in India and showed improved learning using spaced repetition among undergraduate students [23, 24]. However, unfortunately, no study has been conducted in Pakistan, a country with a significantly increasing number of medical students and physicians produced per year [25]. In Pakistan, medical education in medical universities primarily relies on traditional methods of teaching, such as using medical books and lectures. Although these methods may be good learning tools, medical colleges should integrate spaced repetition into their curriculum to make teaching and learning more effective. In a low- to middle-income country such as Pakistan, using premade digital flashcard decks for various subjects may be a more cost-effective learning tool for medical students than buying expensive medical books to obtain the same information. Furthermore, spaced repetition can also be more time efficient during revision periods since it can help students focus more on key points and revise weak areas rather than the information the student already knows well.

In our study, we also identified specific pediatric topics that are difficult to recall in a clinical setting but are undoubtedly crucial to remember, such as developmental milestones, malnutrition, immunization, and IMNCIs. Our results showed that students using traditional learning methods struggled more to retain this information, as reflected in their posttest assessment scores. Similarly, a cross-sectional study conducted at Sultan Qaboos University, Muscat. Studies on the perceptions of undergraduate medical students toward Integrated Management of Childhood Illness (IMCI) Preservice Education have shown that five-year-old medical students need more knowledge regarding IMNCIs [26]. Another cross-sectional study aimed at assessing the nutritional knowledge and attitudes of medical students at King Abdul-Aziz University, Jeddah, Saudi Arabia, demonstrated that participants needed better knowledge regarding malnutrition in children [27]. These findings indicate that medical students struggle to retain critical yet factual concepts related to pediatric health, which will ultimately affect their performance in their clinical rotations and future training. Using improved learning techniques, such as spaced repetition, will aid in retaining minute yet important information and can produce more competent physicians who recognize and promptly address childhood illnesses, improving underfive childhood mortality.

The strengths of our study are that it is the first study of its kind on the use of spaced repetition from Pakistan and the first study of undergraduate medical students studying paediatric medicine. Our sample size included the entire class. An expert panel also validated our assessment content and questions. Both the intervention and control groups were given the same content to study (although in different forms) to ensure fairness in the knowledge provided. We used WhatsApp group chats to ensure that the intervention group completely understood how to use the new learning technology, with daily feedback on several cards reviewed to ensure the completion of cards by the intervention group. Our study also employed pretest and posttest assessments, which enabled us to determine the difference between the two groups under similar testing environments. The MCQs used in the assessment not only involved factual recall but also included critical thinking and problem-solving skills.

Our most significant limitation is that this was a single-institute study, and we could not include a larger sample size, which could have led to a more robust analysis. Furthermore, if we extended the time for learning for more than four weeks, we may have shown improved assessment outcomes. Another limitation of our study is that due to time constraints, we could not study the long-term effects of using spaced repetition on final summative or end-of-year exams. Another limitation was that the students were informed that the assessment scores of the pretest and posttest assessments were formative only. A qualitative aspect of this study could have been included in which students reported their perception of the effectiveness of their respective learning tools. It was also almost impossible to ensure that the intervention group students would not share the flashcards with their friends from the control group. In addition, we did not assess clinical skills using spaced repetition and tested only clinical knowledge.

## Conclusion

Currently, there is more literature about the use of spaced repetition in undergraduate medical students, especially in South Asia. Our study, which was conducted in an undergraduate medical program in Pakistan, showed that spaced repetition via Anki flashcards resulted in significantly higher scores for medical students studying paediatrics than for students using more traditional learning methods, compromising medical books and lectures. Considering that medical students need to retain a vast amount of information, using spaced repetition through flashcards can be a more effective learning tool and is more cost-effective and time-efficient than traditional learning methods.

### Electronic supplementary material

Below is the link to the electronic supplementary material.

Supplementary Material 1

Supplementary Material 2

Supplementary Material 3

Supplementary Material 4

Supplementary Material 5

Supplementary Material 6

## Data Availability

The datasets used and analysed during the current study are available from the corresponding author upon reasonable request.
