# Supplementary material for: Effectiveness of spaced repetition for clinical problem solving amongst undergraduate medical students studying paediatrics in Pakistan
Source: BMC Med Educ. 2024 Jun 18;24:676. doi: 10.1186/s12909-024-05479-y (PMC11186069; doi:10.1186/s12909-024-05479-y)
Supplement: Supplementary file 1 — Supplementary Material 1 [file 12909_2024_5479_MOESM1_ESM.docx]

**Additional File 4-** **Validation of topics and relative weightages (Experts comments)**

| Topic | Strongly Disagree  1 | Disagree  2 | Agree  3 | Strongly Agree  4 | Suggestions |
| --- | --- | --- | --- | --- | --- |
| Developmental Milestones | | | | | |
| Weightage 40% |  | 1 | 4 |  | Expert 1: No comments  Expert 2: It should be 25%. It is important but IMNCI and Malnutrition are vital for Final Year students  Expert 3 No comments  Expert 4:General comments at the end  Expert 5:General comments at the end |
| Learning Objectives |  |  | 5 |  | Expert 1:  Rethink the verb formulate in the objectives. Formulate is usually used for management plan  Expert 2: Enumerate should be used in place of formulate  Expert 3 No comments  Expert 4:General comments at the end  Expert 5:General comments at the end |
| Childhood Vaccination and Immunization | | | | | |
| Weightage 10% |  | 1 | 2 | 2 | Expert 1: No comment  Expert 2: Immunization and vaccination is not same. vaccination should carry 10 %  Expert 3 No comments  Expert 4:General comments at the end  Expert 5:General comments at the end |
| Learning Objectives |  |  | 5 |  | Expert 1: Add catch up vaccination  Expert 2: Discuss should be used for point #3.  Enumerate /discuss for point #5  Expert 3: An objective regarding catchup vaccination can be added though.  Expert 4:  General comments at the end  Expert 5:General comments at the end |
| IMNCI | | | | | |
| Weightage 40% |  |  | 4 | 1 | Expert 1: No comment  Expert 2 : It should be 35% as final year student should be sound in IMNCI  Expert 3 No comments  Expert 4:General comments at the end  Expert 5:General comments at the end |
| Learning Objectives |  |  | 5 |  | Expert 1: No comment  Expert 2 Need more detailing  Expert 3 No comments  Expert 4:General comments at the end  Expert 5-general comments at the end |
| Malnutrition | | | | | |
| Weightage 10% |  |  | 4 | 1 | Expert 1: No comments  Expert 2:  It’s the major issue for our country should be 30 %  Expert 3 No comments  Expert 4:  General comments at the end  Expert 5-general comments at the end |
| Learning Objectives |  | 1 | 4 |  | Expert 1; No comments  Expert 2:Define for point #1  Discuss for point #2 and enumerate for point #3  Classification of malnutrition should be separate point. Discuss management of PCM.  Hypervitaminosis should be deleted.  Expert 3 No comments  Expert 4:General comments at the end  Expert 5:General comments at the end |
| Any other Comments | Expert 4: As you have a specific objective then I agree the weight age is according to your requirements.  Expert 5: You have almost covered all the learning objectives. I am giving few suggestions to increase the scope of the topics.   - You may add name of few milestone scales like Bayley scale in “Growth & development section”. - You may mention rickets in micronutrient section as it is very rampant among our children. | | | | |
