# Supplementary material for: Effectiveness of spaced repetition for clinical problem solving amongst undergraduate medical students studying paediatrics in Pakistan
Source: BMC Med Educ. 2024 Jun 18;24:676. doi: 10.1186/s12909-024-05479-y (PMC11186069; doi:10.1186/s12909-024-05479-y)
Supplement: Supplementary file 2 — Supplementary Material 2 [file 12909_2024_5479_MOESM2_ESM.docx]

**Table 1. Comparison of participant characteristics and test results between control and intervention groups**

| **Test Results** | **All Participants**  **(n= 115)** | **Control Group**  **(n = 45)** | **Intervention Group**  **(n=70)** | **p-value** |
| --- | --- | --- | --- | --- |
| Pre-test (Mean +SD) | 27.94 + 4.18 | 27.96 + 3.69 | 27.93 + 4.53 | p=0.973 |
| Post-test (Mean +SD) | 29.41 + 5.04 | 27.22 + 5.02 | 30.86 + 4.56 | **p <0.001** |

**Table 2. Comparison of pre-test and post-test results within participant groups**

| **Participants** | **Pre-test Result**  **(Mean +/- SD)** | **Post-test Result**  **(Mean +/- SD)** | **p-value** |
| --- | --- | --- | --- |
| All Participants (n=155) | 27.94 + 4.18 | 29.41 + 5.04 | - |
| Control group (n=45) | 27.96 + 3.69 | 27.22 + 5.02 | 0.275 |
| Intervention group (n=70) | 27.93 + 4.53 | 30.86 + 4.56 | **<0.01** |

**Additional File 1-** **Table of specifications (TOS) with relative weightages**

Topic with Exam weightage

1. Developmental milestones 40 % (20)
2. Immunization 10 (5)
3. IMNCI 40% (20)
4. Malnutrition 10% (5)

**Learning objectives:**

**The curriculum of Paediatrics is based on common health-related problems of children in Pakistan. A medical student should have the minimum knowledge and skills to provide comprehensive health care to children and counsel mothers and caregivers on immunization developmental milestones, nutrition, and risk factors. Medical students should be sensitive to the needs and behavior of the children.**

**Immunization:**

**Discuss the principles of immunization**

**Enlist the key points of vaccine preservation and cold chain management**

**Enlist types, contents, efficacy, storage, dose, site, route, contra-indications, and adverse reactions of vaccines – BCG, DPT, OPV, Measles, MMR, hepatitis B, Penta, and Typhoid·**

**Assess the AFP (Acute Flaccid Paralysis) surveillance ·**

**Enlist the types of polio vaccines and herd immunity.**

**Assess the knowledge about special vaccines like Typhoid, Hepatitis A, Chicken pox, Meningococcal, and Rabies.**

**Discuss the diseases and vaccine coverage by the Extended Program of Immunization (EPI).**

**Growth and development**

**Assess the knowledge about standard growth monitoring and growth charts.**

**Assess anthropometry – measurement, and interpretation of weight, length/height, head circumference, and mid-arm circumference. Use of weighing machines and infantometer. Measurement and interpretation of sitting height, US: LS ratio, and arm span.**

**Discuss the abnormalities in growth and development.**

**Discuss different milestones of development and detection of developmental abnormalities. Important milestones in infancy and early childhood are Gross Motor, Fine Motor, Language, and Personal–social development. 3-4 milestones in each developmental field, age of normal appearance, and the upper age of routine psychological and behavioral problems.**

**Integrated Management of Childhood Illness IMNCI**

**Discuss the Integrated Management of Childhood Illness (IMCI) and its role in the preventive and social aspects of pediatrics.**

**4. Nutrition**

**Enlist the standard nutritional requirements (breastfeeding, infant feeding, weaning).**

**Enumerate the nutritional disorders (malnutrition, rickets, scurvy, Vitamin A deficiency, iodine deficiency, and iron deficiency)**

**Enlist various vitamin deficiencies (vitamins A, B, C, D, E, K ). State the recommended daily allowances.**

**Enumerate the causes and management of malnutrition and its classification; identify the risk factors.**

**Discuss the management of protein-calorie malnutrition as per WHO guidelines.**

**Enlist the micronutrient deficiencies and their management (iron, zinc, biotin)**

**Additional File 2- Expert panel**

| Serial number | Expert name, Institute, qualification, years of experience | Accepted, did not respond, regret |
| --- | --- | --- |
| 1. | Dr.Shakeel Ahmed  BUMDC  FCPS FRCPCH, DIPLOMA in HPE (AKU)  25 years plus | Accepted |
| 2. | Dr.Ammara Hameed  BUMDC  FCPS (ISC in HPE)  5 years plus | Accepted |
| 3. | Dr. Arshalooz  AKUH  FCPS, MHPE  20 years Plus | Accepted but did not respond later |
| 4. | Dr. Danish Abdul Aziz  AKUH  FCPS, MHPE  10 years plus | Did not respond |
| 5. | Dr. Sana Saeed  AKUH  FCPS, MHPE  10 years plus | Accepted |
| 6. | Dr Sadaf Saeed  Indus hospital  FCPS, MHPE  10 years plus | Accepted |
| 7. | DR.Maria Zahoor  NICH  MCPS, MSC , MRCPCH part 1  10 years | Regret |
| 8. | Dr. Shireen Bham  FMC  FCPS,  12 years | Accepted |

**Additional File 3- Tool for Validation of topics and relative weightages**

| Topic | Strongly Disagree  1 | Disagree  2 | Agree  3 | Strongly Agree  4 | Suggestions | |
| --- | --- | --- | --- | --- | --- | --- |
| Developmental Milestones | | | | | | |
| Weightage 40% |  |  |  |  |  | |
| Learning Objectives |  |  |  |  |  | |
| Childhood Vaccination and Immunization | | | | | | |
| Weightage 10% |  |  |  |  |  | |
| Learning Objectives |  |  |  |  |  | |
| IMNCI | | | | | | |
| Weightage 40% |  |  |  |  |  | |
| Learning Objectives |  |  |  |  |  | |
| Malnutrition | | | | | | |
| Weightage 10% |  |  |  |  |  |  |
| Learning Objectives |  |  |  |  |  |  |

**Additional File 4-** **Validation of topics and relative weightages (Experts comments)**

| Topic | Strongly Disagree  1 | Disagree  2 | Agree  3 | Strongly Agree  4 | Suggestions |
| --- | --- | --- | --- | --- | --- |
| Developmental Milestones | | | | | |
| Weightage 40% |  | 1 | 4 |  | Expert 1: No comments  Expert 2: It should be 25%. It is important but IMNCI and Malnutrition are vital for Final Year students  Expert 3 No comments  Expert 4:General comments at the end  Expert 5:General comments at the end |
| Learning Objectives |  |  | 5 |  | Expert 1:  Rethink the verb formulate in the objectives. Formulate is usually used for management plan  Expert 2: Enumerate should be used in place of formulate  Expert 3 No comments  Expert 4:General comments at the end  Expert 5:General comments at the end |
| Childhood Vaccination and Immunization | | | | | |
| Weightage 10% |  | 1 | 2 | 2 | Expert 1: No comment  Expert 2: Immunization and vaccination is not same. vaccination should carry 10 %  Expert 3 No comments  Expert 4:General comments at the end  Expert 5:General comments at the end |
| Learning Objectives |  |  | 5 |  | Expert 1: Add catch up vaccination  Expert 2: Discuss should be used for point #3.  Enumerate /discuss for point #5  Expert 3: An objective regarding catchup vaccination can be added though.  Expert 4:  General comments at the end  Expert 5:General comments at the end |
| IMNCI | | | | | |
| Weightage 40% |  |  | 4 | 1 | Expert 1: No comment  Expert 2 : It should be 35% as final year student should be sound in IMNCI  Expert 3 No comments  Expert 4:General comments at the end  Expert 5:General comments at the end |
| Learning Objectives |  |  | 5 |  | Expert 1: No comment  Expert 2 Need more detailing  Expert 3 No comments  Expert 4:General comments at the end  Expert 5-general comments at the end |
| Malnutrition | | | | | |
| Weightage 10% |  |  | 4 | 1 | Expert 1: No comments  Expert 2:  It’s the major issue for our country should be 30 %  Expert 3 No comments  Expert 4:  General comments at the end  Expert 5-general comments at the end |
| Learning Objectives |  | 1 | 4 |  | Expert 1; No comments  Expert 2:Define for point #1  Discuss for point #2 and enumerate for point #3  Classification of malnutrition should be separate point. Discuss management of PCM.  Hypervitaminosis should be deleted.  Expert 3 No comments  Expert 4:General comments at the end  Expert 5:General comments at the end |
| Any other Comments | Expert 4: As you have a specific objective then I agree the weight age is according to your requirements.  Expert 5: You have almost covered all the learning objectives. I am giving few suggestions to increase the scope of the topics.   - You may add name of few milestone scales like Bayley scale in “Growth & development section”. - You may mention rickets in micronutrient section as it is very rampant among our children. | | | | |
