# Supplementary material for: Effectiveness of spaced repetition for clinical problem solving amongst undergraduate medical students studying paediatrics in Pakistan
Source: BMC Med Educ. 2024 Jun 18;24:676. doi: 10.1186/s12909-024-05479-y (PMC11186069; doi:10.1186/s12909-024-05479-y)
Supplement: Supplementary file 4 — Supplementary Material 4 [file 12909_2024_5479_MOESM4_ESM.docx]

**ANNEXURE 1.**

**Additional File 1-** **Table of specifications (TOS) with relative weightages**

Topic with Exam weightage

1. Developmental milestones 40 % (20)
2. Immunization 10 (5)
3. IMNCI 40% (20)
4. Malnutrition 10% (5)

**Learning objectives:**

**The curriculum of Paediatrics is based on common health-related problems of children in Pakistan. A medical student should have the minimum knowledge and skills to provide comprehensive health care to children and counsel mothers and caregivers on immunization developmental milestones, nutrition, and risk factors. Medical students should be sensitive to the needs and behavior of the children.**

**Immunization:**

**Discuss the principles of immunization**

**Enlist the key points of vaccine preservation and cold chain management**

**Enlist types, contents, efficacy, storage, dose, site, route, contra-indications, and adverse reactions of vaccines – BCG, DPT, OPV, Measles, MMR, hepatitis B, Penta, and Typhoid·**

**Assess the AFP (Acute Flaccid Paralysis) surveillance ·**

**Enlist the types of polio vaccines and herd immunity.**

**Assess the knowledge about special vaccines like Typhoid, Hepatitis A, Chicken pox, Meningococcal, and Rabies.**

**Discuss the diseases and vaccine coverage by the Extended Program of Immunization (EPI).**

**Growth and development**

**Assess the knowledge about standard growth monitoring and growth charts.**

**Assess anthropometry – measurement, and interpretation of weight, length/height, head circumference, and mid-arm circumference. Use of weighing machines and infantometer. Measurement and interpretation of sitting height, US: LS ratio, and arm span.**

**Discuss the abnormalities in growth and development.**

**Discuss different milestones of development and detection of developmental abnormalities. Important milestones in infancy and early childhood are Gross Motor, Fine Motor, Language, and Personal–social development. 3-4 milestones in each developmental field, age of normal appearance, and the upper age of routine psychological and behavioral problems.**

**Integrated Management of Childhood Illness IMNCI**

**Discuss the Integrated Management of Childhood Illness (IMCI) and its role in the preventive and social aspects of pediatrics.**

**4. Nutrition**

**Enlist the standard nutritional requirements (breastfeeding, infant feeding, weaning).**

**Enumerate the nutritional disorders (malnutrition, rickets, scurvy, Vitamin A deficiency, iodine deficiency, and iron deficiency)**

**Enlist various vitamin deficiencies (vitamins A, B, C, D, E, K ). State the recommended daily allowances.**

**Enumerate the causes and management of malnutrition and its classification; identify the risk factors.**

**Discuss the management of protein-calorie malnutrition as per WHO guidelines.**

**Enlist the micronutrient deficiencies and their management (iron, zinc, biotin)**
