# Supplementary material for: Effectiveness of spaced repetition for clinical problem solving amongst undergraduate medical students studying paediatrics in Pakistan
Source: BMC Med Educ. 2024 Jun 18;24:676. doi: 10.1186/s12909-024-05479-y (PMC11186069; doi:10.1186/s12909-024-05479-y)
Supplement: Supplementary file 5 — Supplementary Material 5 [file 12909_2024_5479_MOESM5_ESM.docx]

**ANNEXURE 3:**

**Additional File 3- Tool for Validation of topics and relative weightages**

| Topic | Strongly Disagree  1 | Disagree  2 | Agree  3 | Strongly Agree  4 | Suggestions | |
| --- | --- | --- | --- | --- | --- | --- |
| Developmental Milestones | | | | | | |
| Weightage 40% |  |  |  |  |  | |
| Learning Objectives |  |  |  |  |  | |
| Childhood Vaccination and Immunization | | | | | | |
| Weightage 10% |  |  |  |  |  | |
| Learning Objectives |  |  |  |  |  | |
| IMNCI | | | | | | |
| Weightage 40% |  |  |  |  |  | |
| Learning Objectives |  |  |  |  |  | |
| Malnutrition | | | | | | |
| Weightage 10% |  |  |  |  |  |  |
| Learning Objectives |  |  |  |  |  |  |
