# Supplementary material for: Effectiveness of spaced repetition for clinical problem solving amongst undergraduate medical students studying paediatrics in Pakistan
Source: BMC Med Educ. 2024 Jun 18;24:676. doi: 10.1186/s12909-024-05479-y (PMC11186069; doi:10.1186/s12909-024-05479-y)
Supplement: Supplementary file 6 — Supplementary Material 6 [file 12909_2024_5479_MOESM6_ESM.doc]

**Informed Consent**

|  |  | **Project Information** | | |  |
| --- | --- | --- | --- | --- | --- |
|  |  |  |  |  |  |
|  | Project Title:  Effectiveness of Spaced Repetition for Clinical Problem solving amongst Pediatric students in Pakistan |  |  | Project Number: 6175 |  |
|  |  |  |  |  |  |
|  |  |  |  |  |  |
|  | ERC Ref No: ERC 69/2020 |  |  | Sponsor: none |  |
|  |  |  |  |  |  |
|  |  |  |  |  |  |
|  | Principal Investigator: | Dr.Naveed Yousuf |  | Organization: AKU Medical College |  |
|  |  |  |  |  |  |
|  |  |  |  |  |  |
|  | Location: Karachi |  |  | Phone: 03009282250 |  |
|  |  |  |  |  |  |
|  |  |  |  |  |  |
|  | Other Investigators: Dr.Rahila Ali Mazhar  Dr.Shazia Fakhir Durrani |  |  | Organization: Bahria University Medical and Dental college |  |
|  |  |  |  |  |  |
|  |  |  |  |  |  |
|  | Location: Karachi |  |  | Phone: 03002263604 |  |
|  |  |  |  |  |  |
|  |  |  |  |  |  |

“I am Dr Shazia Fakhir Durrani from Department of Paediatrics, Bahria University Medial and Dental College and doing a research on ’**Effectiveness of Spaced Repetition for Clinical Problem solving amongst Paediatric students in Pakistan**”

Medical students need an efficient self-study method which would help them to memorize and retain knowledge for quick application and problem solving.

Spaced repetition learning methods are comparatively a new concept in medical education, not more than 10 years and may involve the use of flash cards which appear at a space interval of time and help in learning. This method retains information in long term memory for future recall.

**Spaced repetition** builds mastery and helps remember and apply what you have learned. It locks key information into long term memory for peak performance.

1. **PURPOSE OF THIS RESEARCH STUDY**

You are being asked to participate in a research study designed to assess spaced repetition framework and see if it is better in retaining information in terms of knowledge and its application and problem solving.

1. **PROCEDURES**

You will be asked to take a pretest which will include 50 multiple choice questions involving problem solving cases related to pediatrics.

Then class will be divided randomly into 2 groups. One will get enrolled in a website including spaced repetition flash cards for the topics included in the test. Review time will be 4 weeks.

Post -test will be taken after 4 weeks.

Second control group will study the same topic for a period of 4 weeks from their textbooks and notes, as they usually do without spaced repetition and as massed learning.

This will be for research purpose only and pre and post test results will be formative only,

The purpose of the research is to study the effectiveness of spaced repetition learning method as compared to the massed learning method and will not have any effect on your summative assessments.

Time involved will be total of 6 weeks

Intervention will be Quasi experiment

1. **POSSIBLE RISKS OR DISCOMFORT**

There will be no risk of physical injury, nor possible psychological, social or economic harm or discomfort.

1. **POSSIBLE BENEFITS**

This will directly benefit students and their long term learning and problem solving skills and benefit medical education.

1. **FINANCIAL CONSIDERATIONS**

There is no financial compensation for your participation in this research.

You will be needing your own WiFi and computers

1. **CONFIDENTIALITY**

ERC approval will be taken from both the institutes.

Written informed consent shall be taken from all students. The written document with informed consent will be signed by the students and the investigator. One copy of the consent form will be given to the student.

Students will be given an informed choice for participation and names and results will not be disclosed to anyone else except the student himself/herself and or used in any final/summative assessment.

Names will be kept anonymous. They will be non-identifiable and coding will be used to mask names. No one will have access to data except the reviewer.

All hard copies will be kept in a locked file and the data on the computers will be password protected.

Software flash cards will be provided to all the students in the intervention group after the post test is completed, to ensure that the~~y~~ students will have enough time to study the flash cards before the post quiz and the control group will get the same flash cards after the post test and be given enough time to study them before their summative assessments.

However, any records or data obtained as a result of your participation in this study may be inspected by the sponsor or by AKU ERC members.

.

1. **RIGHT TO REFUSE OR WITHDRAW**

You are free to choose whether or not to participate in this study. There will be no penalty or loss of benefits to which you are otherwise entitled if you choose not to participate. You will be provided with any significant new findings developed during the course of this study that may relate to or influence your willingness to continue participation. In the event you decide to discontinue your participation in the study.

These are the potential consequences that may result.

Please notify

Dr. Shazia Fakhir

03002263604

of your decision so that your participation can be orderly terminated.

In addition, your participation in the study may be terminated by the investigator without your consent under the following circumstances.

It may be necessary for the sponsor of the study to terminate the study without prior notice to, or consent of, the participants in the event that there is loss of finding.

1. **AVAILABLE SOURCES OF INFORMATION**

Any further questions you have about this study will be answered by the Principal Investigator:

Name: Dr.Naveed Yousuf

Phone Number: 03009282250

Any questions you may have about your rights as a research subject will be answered by:

Name: : Dr.Naveed Yousuf

Phone Number: 03009282250

:

In case of a research-related emergency, call: Day Emergency Number:03002263604

Night Emergency Number: 03002263604

1. **AUTHORIZATION**

I have read and understand this consent form, and I volunteer to participate in this research study. I understand that I will receive a copy of this form. I voluntarily choose to participate, but I understand that my consent does not take away any legal rights in the case of negligence or other legal fault of anyone who is involved in this study.

Name of participant

Date:

Signature of participant:

Date:

3

Signature of Principal Investigator:

Date:

Name and Signature of person obtaining consent:

Date:

4
